# Immediate Fluoxetine Hypersensitivity With Predominant Airway and Oropharyngeal Symptoms in an Adolescent

**DOI:** 10.1155/crpe/6833218

**Published:** 2026-08-17

**Authors:** Mariana Viegas, Jacinta Mendes, Sónia Lemos, Estefânia Maia, Gina Rubino

**Affiliations:** ^1^ Oeste Local Health Unit, Caldas da Rainha, Portugal; ^2^ Pediatric Allergology Consultation, Ambulatory Pediatric Department, Coimbra Local Health Unit, Coimbra´s Pediatric Hospital, Coimbra, Portugal

**Keywords:** basophil activation test, case report, drug allergy, fluoxetine, hypersensitivity

## Abstract

Fluoxetine is a widely prescribed selective serotonin reuptake inhibitor in children and adolescents. Hypersensitivity reactions are rare and poorly characterized in this population. We report the case of a 17‐year‐old female who developed acute throat tightness with perceived oropharyngeal swelling and respiratory symptoms within 30 minutes of the first dose of fluoxetine, in the absence of cutaneous manifestations. Symptoms improved with bronchodilator therapy. Basophil activation testing demonstrated drug‐induced activation, and an oral drug provocation test reproduced symptoms, confirming hypersensitivity. Previously reported pediatric cases included delayed urticaria, angioedema, and drug rash with eosinophilia and systemic symptoms. In contrast, this case highlights an immediate reaction predominantly affecting the airway. This report expands the known clinical spectrum of fluoxetine hypersensitivity reactions and emphasizes that the absence of cutaneous manifestations does not exclude drug allergy.

## 1. Introduction

Fluoxetine is a selective serotonin reuptake inhibitor (SSRI) widely used in adolescents for depressive and anxiety disorders [1–3]. Hypersensitivity reactions to fluoxetine are rare, and their underlying immunopathogenic mechanisms remain poorly understood. Proposed mechanisms include immediate hypersensitivity reactions mediated by mast cell and basophil activation, as well as delayed T‐cell–mediated reactions, although definitive immunological pathways have not been established [4, 5]. Previously reported pediatric cases describe predominantly cutaneous or delayed phenotypes [6–8]. We report a drug‐provocation–confirmed immediate drug hypersensitivity reaction presenting predominantly with respiratory symptoms. Recognition of atypical immediate presentations is clinically important because symptoms may be misattributed to the underlying psychiatric disorder.

## 2. Case Report

A 17‐year‐old female followed in Child and Adolescent Psychiatry for depressive disorder and generalized anxiety presented to the pediatric emergency department with oropharyngeal discomfort and perceived swelling accompanied by vomiting approximately 30 minutes after ingestion of her first 20‐mg dose of fluoxetine, prescribed after switching from sertraline, which had been well tolerated. She denied pruritus, urticaria, or other cutaneous symptoms. Her past medical history was unremarkable, with no known drug allergies, asthma, or atopic disease. She also had no history of food allergy. No unusual foods or other potential allergenic exposures had been consumed immediately prior to symptom onset, making alternative causes for the reaction unlikely.

Approximately two hours after drug intake, she exhibited noisy breathing and mild respiratory distress with normal peripheral oxygen saturation. Pulmonary auscultation revealed inspiratory wheezing. Although the patient reported a sensation of oropharyngeal swelling, objective swelling was not documented on physical examination. No urticaria, angioedema, mucosal lesions, or skin rash were observed. Cardiovascular examination was unremarkable, with stable hemodynamic parameters, and the remainder of the physical examination was normal. She was treated with inhaled salbutamol with rapid symptom resolution and discharged after observation. Serum tryptase was not measured during the acute episode.

The patient was referred for allergological evaluation. Given the temporal relationship with drug intake, a reaction compatible with an immediate drug hypersensitivity reaction was suspected. A basophil activation test performed three months later demonstrated low‐level (4.2%) basophil activation in response to fluoxetine at a 1:10 dilution, whereas no activation was observed at lower concentrations or in the negative control. Positive controls were adequate. Given the absence of validated diagnostic thresholds for fluoxetine, the BAT result was considered supportive but not diagnostic and was interpreted in conjunction with the clinical history and the subsequent drug provocation test.

Five months after the initial reaction, an oral drug provocation test was performed in a controlled hospital setting, as drug provocation testing remains the diagnostic gold standard when safely feasible [7]. Fluoxetine was administered as a divided tablet. Shortly after administration of the 5‐mg dose of fluoxetine, administered with 30‐minute observation interval, the patient developed throat tightness, nausea, dizziness, and fatigue, without cutaneous manifestations. Vital signs remained stable, and pulmonary auscultation was normal. The test was considered positive and discontinued, with gradual symptom resolution after oral antihistamine treatment. No delayed reactions were observed during follow‐up. The sequence of events is summarized as follows: initial reaction within 30 minutes of drug intake, allergological evaluation at 3 months, and drug provocation test at 5 months. A diagnosis of immediate drug hypersensitivity reaction to fluoxetine was confirmed by drug provocation testing. Written informed consent was obtained from the patient and her legal guardians. According to institutional policy, ethics committee approval was not required for a single clinical case. This case report was prepared in accordance with the CARE guidelines.

## 3. Discussion

Published pediatric reports describe heterogeneous fluoxetine hypersensitivity phenotypes ranging from delayed DRESS to urticaria and angioedema [6–8]. In contrast, our patient presented with an early reaction characterized predominantly by upper airway and respiratory symptoms without cutaneous involvement, a presentation not emphasized in previous pediatric descriptions (Table 1).

**TABLE 1:** Published pediatric cases of fluoxetine drug hypersensitivity reactions.

Author (year)	Age	Latency	Clinical presentation	Type of reaction
Vignesh et al. (2017)	4 years	Weeks	Rash, eosinophilia, systemic involvement	Nonimmediate DHR (DRESS)
Adanır et al. (2018)	11 years	Hours–days	Urticaria, angioedema	Immediate DHR (urticaria/angioedema)
Muzenda et al. (2023)	Adolescent	1 month	Late‐onset urticaria	Nonimmediate DHR (delayed urticaria)
Current case	17 years	30 minutes	Throat tightness, vomiting, respiratory symptoms	Immediate DHR (respiratory‐predominant, noncutaneous)

Abbreviations: DHR, drug hypersensitivity reaction; DRESS, drug rash with eosinophilia and systemic symptoms.

The short latency after first exposure and reproducibility during drug provocation are consistent with an immediate drug hypersensitivity reaction according to current classification concepts, although an IgE‐mediated mechanism cannot be confirmed [4, 5].

The absence of mucocutaneous signs may hinder recognition, particularly in adolescents receiving psychotropic medication, and symptoms may be misinterpreted as anxiety, globus sensation, or functional respiratory complaints. Although immediate reactions often suggest IgE‐mediated mechanisms, drug‐specific IgE to fluoxetine has not been demonstrated, and the underlying mechanism remains uncertain; non–IgE‐mediated pathways, including direct mast cell activation, are plausible [5].

Drug provocation testing remains the diagnostic gold standard for diagnosis when safely feasible according to international guidelines [4]. In vitro tests such as basophil activation testing may provide adjunctive information in drug hypersensitivity reactions; however, no validated diagnostic cutoff exists for fluoxetine, and results must be interpreted within the clinical context. The observed activation level was low and would be considered borderline according to commonly used BAT criteria for other drugs; therefore, BAT was regarded only as a supportive investigation. Recognition of this phenotype is clinically relevant because selective SSRIs are widely prescribed in adolescents [2, 3], and misinterpretation may lead to unintended reexposure to the culprit drug.

Therefore, this case is best classified as an immediate drug hypersensitivity reaction with an uncertain immunopathological mechanism.

This case emphasizes that immediate hypersensitivity reactions to fluoxetine may occur without cutaneous manifestations, potentially delaying recognition. This presentation may be underrecognized in clinical practice.

## 4. Conclusion

Immediate respiratory reactions to fluoxetine may occur without cutaneous manifestations and may mimic anxiety‐related symptoms. Drug provocation testing remains the reference standard for diagnosis when it can be safely performed, preventing incorrect labeling or unsafe reexposure. Clinicians should consider drug hypersensitivity reactions even when presentation is exclusively respiratory, particularly in adolescents receiving psychotropic therapy. Awareness of this presentation is essential for safe prescribing in pediatric psychiatric practice.

## Funding

No funding was received for this manuscript.

## Consent

Patient consent was obtained.

## Conflicts of Interest

The authors declare no conflicts of interest.

## Data Availability

The data that support the findings of this study are available on request from the corresponding author. The data are not publicly available due to privacy or ethical restrictions.
